# Editorial: Ethylene: A Key Regulatory Molecule in Plants

**DOI:** 10.3389/fpls.2017.01782

**Published:** 2017-10-16

**Authors:** Nafees A. Khan, M. I. R. Khan, Antonio Ferrante, Peter Poor

**Affiliations:** ^1^Department of Botany, Aligarh Muslim University, Aligarh, India; ^2^Crop and Environmental Sciences Division, International Rice Research Institute, Metro Manila, Philippines; ^3^Department of Agricultural and Environmental Sciences, Università degli Studi di Milano, Milan, Italy; ^4^Department of Plant Biology, University of Szeged, Szeged, Hungary

**Keywords:** ethylene, physiology, metaboilsm, phytohormones, signaling molecules

Ethylene is a simple gaseous phytohormone with multiple roles in regulation of metabolism at cellular, molecular, and whole plant level (Pierik et al., 2006; Lin et al., 2009; Schaller, 2012; Khan N. A. and Khan M. I. R., 2014). It influences performance of plants under optimal and stressful environments by interacting with other signaling molecules (Müller and Munné-Bosch, 2015; Thao et al., 2015). The action of ethylene depends on its concentration in cell and the sensitivity of plants to the hormone (Pierik et al., 2006; Habben et al., 2014; Arraes et al., 2015; Sun et al., 2016). In recent years, research on ethylene has been focused due to its dual action on the regulation of plant processes at physiological and molecular level. The aim of the current research topic was to explore and update our understanding on its regulatory role of ethylene in plant developmental mechanisms at cellular or whole plant level under optimal and changing environmental conditions. The present edited volume includes original research papers and reviews articles describing ethylene's regulatory role in plant development during plant ontogeny and how it interacts with biotic and abiotic stress factors. For better understanding of the articles included in this volume, papers have been grouped into three categories.

## Role of ethylene in developmental process

### Flower development and fruit ripening

The development of plants is well regulated by several processes working co-ordinately. This involves hormonal regulation in synchronization with other processes. Phytohormones influence plant development more precisely acting as a signaling molecule. The review article by Iqbal et al. emphasized the ethylene's roles in growth, development and senescence of leaves, flowers, and fruits. The ethylene controls longevity of plants that depends on ethylene level, its perception, and the hormonal crosstalk. It has been found that exogenously applied ethylene up-regulated *PhEIL2* gene and down-regulated *PhEIL3* gene, the genes responsible for flower senescence. Functional analysis of these two genes using VIGS system demonstrated that by silencing both *PhEIL2* and *PhEIL3* genes the flower life was significantly extended. In a study on silenced petunia lines, it was found that the expression of *PhERF3* and *PhCP2* genes was associated with ethylene production (Liu et al.). The EIN3 is an important transcription factor that regulates primary plant response to ethylene. The EIN3 regulates the gene expression by different DNA-binding sequences in the gene promoters. Results of the study on *EIN3/EIL1* binding sites and chromatin states in *Arabidopsis thaliana* showed that the chromatin state 4 was particularly important in regulation of plant response to ethylene. The homolog of *EIN3* of *Arabidopsis* in *Petunia* is the *EIL* gene and its role has been studied in flower senescence. The data confirms that EIN3 is the most important transcriptional regulator in the ethylene signaling pathway (Zemlyanskaya et al.).

Ethylene Response Factors (ERFs) have been reported to be involved in ethylene signaling and/or ethylene response, but little is known about their roles in fruit ripening. Fan et al. reported that ethylene plays an essential role in fruit ripening via modulation of ethylene signaling pathway by identifying DREB transcription factor with EAR motif, designated as *MaDEAR1*. They found that *MaDEAR1* binds to the DRE/CRT motifs in promoters of several cell wall-modifying genes, which repressed their activities and negatively involved in ethylene-mediated ripening of banana fruit. The study of Tranbarger et al. revealed that during fruit ripening of monocotyledonous plants and in particular in *Elaeis guineensis*, the ethylene induced cell wall and middle lamella expansion and degradation. This transition was regulated by different transcription factors some of them under ethylene regulation. The most important in the fruit ripening of this monocot has been found to be NAC domain transcription factors. Ethylene exposure studies revealed that the most inducible were *EgNAC6* and *EgNAC7* (Tranbarger et al.). The comparison of expression data of these genes with other eudicots could provide useful information on fruit ripening species evolution.

### Growth and nodulation

In this research topic, the interaction of ethylene and light on hypocotyl growth of *A. thaliana* has been reviewed (Yu and Huang). They showed that role of ethylene on hypocotyls growth under light or dark conditions could be ascertained through over-expression of ethylene production or inhibition of ethylene biosynthesis using *Arabidopsis* mutants. In light condition, ethylene induces the expression of *PHYTOCHROME INTERACTING FACTOR 3* (PIF3) and degradation of *ELONGATED HYPOCOTYL 5* (HY5), resulting in hypocotyl growth. In dark, instead, the suppression of hypocotyl development occurs by inducing the *ETHYLENE RESPONSE FACTOR 1* (ERF1) and *WAVE-DAMPENED 5* (WDL5) through the EIN3. This gene is additionally regulated by *CONSTITUTIVE PHOTOMORPHOGENIC 1* (COP1) and phytochrome B (phyB). Plant floral organ abscission is also one of the important developmental processes, which is mediated by ethylene. Wang et al. found that ethylene accelerated the organ abscission in *Arabidopsis* by regulating the expression of *AtDOF4.7* transcription factor and the peptide ligand *INFLORESCENCE DEFICIENT IN ABSCISSION* (*IDA*), which repressed expression of *AtDOF4.7*. MAPK cascades are involved in downstream of *IDA*-mediated abscission pathway. Wang et al. found *in vivo* interaction between MPK3/6 and AtDOF4.7 suggesting that AtDOF4.7 protein levels were regulated by this phosphorylation. Choong et al. showed that temperate crops cannot grow well in the tropics without root zone cooling. They reported that lower ethylene concentrations in root zone corresponded to higher shoot growth at cooler root zone temperatures; the cultivars that were less sensitive could be selected for agricultural purposes. Ma et al. observed that ethylene significantly inhibited postharvest peel browning in pear plants. In this study it was shown that protection of “Huangguan” pear from skin browning was possible through exogenous ethylene application. Genome wide identification and gene expression profiling during legume plant nodulation reveal that ethylene signaling pathway regulates nodulation in soybean (Wang et al.). They identified 11 ethylene receptor family genes in soybean through homology searches. The analysis of their expression patterns showed that these ethylene receptor genes are differentially expressed in various soybean tissues and organs, during rhizobia–host cell interactions and nodulation.

### Interaction of ethylene with other hormones

Liu et al. found an interaction of ethylene with methyl jasmonate (MeJA). They analyzed the phenolic compounds in *Catharanthus roseus* using a non-targeted metabolomics method. There were 34 phenolics, which belonged to 3 categories: 7 C6C1-, 11 C6C3-, and 16 C6C3C6-compounds, in addition to seven other metabolites. Among these compounds, vanillyl alcohol in leaves was elevated 50 times in the presence of ethylene and MeJA. However, in case of C6C3C6- type compounds, ethylene and MeJA presence exhibited an inhibitory effect. Explaining the interaction of ethylene and auxin, Abts et al. observed that the early root growth of sugar beet showed a biphasic ethylene response. The exogenously applied auxin (indole-3-acetic acid; IAA) induced root elongation in sugar beet by stimulating ethylene biosynthesis by redirecting the pool of available ACC toward ethylene instead of malonyl-ACC (MACC). In addition, IAA induced the expression of several *ACS* and *ACO* genes during seedling development suggesting that the general ethylene-auxin cross talk model was different in this plant. Ethylene in coordination with nitric oxide (NO) is also known to influence the cell cycle. Novikova et al. reported that ethylene and NO signaling interacts and plays important role in regulating cell cycle in *Arabidopsis*. They found that cell cycle progression was dependent on NO presence in the cells and on EIN2 (for ethylene production) in *Arabidopsis*.

## Ethylene regulates responses of plants to abiotic stress conditions

Ethylene is regarded as a stress-responsive hormone besides its roles in regulation of plant growth and development (Khan M. I. R. and Khan N. A., 2014). In the current topic, Zapata et al. found that salt shock caused rapid increase in the production of ethylene, ACC and polyamine concentrations both in shoots and roots of the four investigated plant species, which were related in the sensitivity to salt stress. In salt tolerant plants, ethylene production was lower, which was found still higher in the most sensitive one. Moreover, they did not observe any competition between polyamines and ethylene biosynthesis for their common precursor, S-adenosylmethionine (SAM). In tomato, it has been demonstrated that ethylene biosynthesis is influenced by the vigor of rootstock in grafted plants and potassium availability. In particular, results showed that low ACC content was able to improve K^+^ uptake in grafted tomato plants. The effect of ethylene was the outcome of the interaction with other plant hormones. It is considered a negative ethylene regulation since high biosynthesis has been found associated with low tolerance of plants to K^+^ deficiency (Martínez-Andújar et al.). Pan et al. found the role of ethylene in antagonizing salt induced growth retardation and cell death process by transcription controlling of ethylene-BAG and senescence associated genes in *Arabidopsis*. Ethylene and salinity antagonistically controlled BAG family, ethylene, and senescence related genes to alleviate the salt induced cell death. Abozeid et al. reported that ethylene played a role in modulating root morphogenesis under cadmium stress in *A. thaliana* by increase in the activity of SOD isoenzymes. It was noted that ethylene-insensitive mutants (*ein2-5* and *ein3-1eil1-1*) have decreased root growth compared to wild type Col-0 along with increased superoxide concentration in roots of *ein2-5* and *ein3-1, eil1-1*. However, application of exogenous ACC (precursor of ethylene biosynthesis) decreased superoxide accumulation in Col-0 root tips and increased the activity of SOD isoenzymes under Cd stress. Khan et al. found that exogenous application of ethylene with sufficient sulfur level counteracted the cadmium-induced photosynthetic and growth inhibition in mustard plants. They reported that the combined application of ethephon and sulfur synergistically improved photosynthetic performance under the stress condition by reducing oxidative stress, ethylene and glucose sensitivity. On the other hand, the better performance of plants was correlated with increase in cysteine and methionine content and reduced glutathione (GSH) contents. Valluru et al. selected two wheat genotypes as drought-tolerant and drought-sensitive and observed the effect on endogenous ethylene and abscisic acid (ABA). Both the drought tolerant and drought sensitive groups increased endogenous ethylene and ABA concentrations under mild drought condition. Further, they observed that shoot dry weight of the drought tolerant and drought sensitive groups distinctly regulated by specific ABA: ethylene ratio. Application of exogenous ABA and ethylene increased relative growth rate in both groups compared to control with increased carbohydrate content. A review by Li and Lan showed that Fe deficiency developed hindrance in various physiological, morphological, metabolic, and gene expression changes with cellular Fe homeostasis in strategy I plants, and ethylene was involved in Fe deficiency responses of plants. Additionally, the review highlighted a way to find out how ethylene participates in the Fe deficiency response via integration of important genes and proteins, regulated both by Fe deficiency, and ethylene into a systemic network by gene co-expression analysis.

## Ethylene regulates responses of plants to biotic stress conditions

The role of ethylene in plants exposed to biotic stress has been studied in different crops and different diseases. In this research topic, Wang et al. have shown that ethylene plays a pivotal role in the sensitivity to *Alternaria alternata* in sand pear (*Pyrus pyrifolia*). The two cultivars used in the study were Cuiguan (tolerant cultivar) and Sucui1 (sensitive cultivar). High ethylene production induced fungus development, while low ethylene evolution was associated to plant resistance (Wang et al.). In both the cultivars a correlation was found between ethylene biosynthesis and detoxifying enzyme activities. In particular, a close relationship was found between ethylene and catalase (CAT) activity. In sensitive cultivar, it was found that high ethylene biosynthesis associated with high level of hydrogen peroxidase and low CAT activity were the favorable conditions for *A. alternata* development and programmed cell death (PCD) induction. Analogously, it has been shown that ethylene has a primary role in endophytic fungi growth as observed in *Atractylodes lancea*. The endophytic fungus *Gilmaniella* sp. AL12 induced ethylene in *A. lancea* and subsequently the accumulation of sesquiterpenoids. The ethylene seems to play an upstream regulation of sesquiterpenes biosynthesis, interacting with other plant hormones such as jasmonic acid and salicylic acid (Yuan et al.).

Wang et al. showed that ethylene was involved in the susceptibility of maize to *Aspergillus flavus*. Interestingly, both colonization and conidiation of *A. flavus* were reduced in kernels treated with ethylene biosynthesis inhibitor. Surprisingly, kernels of *acs2* and *acs6*, in case of the two ethylene biosynthetic mutants, displayed enhanced seed colonization and conidiation, but without increasing the levels of aflatoxin after the infection. These results suggested that ethylene emitted by infected seeds facilitated colonization by *A. flavus* but without any aflatoxin production. Boex-Fontvieille et al. reported that exogenous application of ethylene precursor ACC and wounding strongly up-regulated the HEC1-dependent Kunitz-protease inhibitor 1 (Kunitz-PI;1) gene expression in apical hook of etiolated *Arabidopsis* seedlings. They summarized that the ethylene-triggered expression of *Kunitz-PI;1* contributed to the protection of seedlings against herbivorous arthropods such as *Porcellioscaber* (woodlouse) and *Armadillidium vulgare* (pillbug), because it can play role in the herbivore deterrence by inhibiting the digestive proteases.

Frontiers research topic provides an excellent platform and opportunity to publish perspective papers in ethylene biology research. Contributed authors significantly attempted a solution for abiotic and biotic stresses tolerance via ethylene manipulation. Additionally, authors also gave a depth insight into the understanding the role of ethylene in growth and development of plants. Altogether, the research topic as presented here documents recent advances in ethylene biology research. In the present volume, numbers of problems from basics to applied scientific knowledge-based questions were addressed and drive plant scientists for a common future goal through this research topic.

## Author contributions

All authors listed have made a substantial, direct and intellectual contribution to the work, and approved it for publication.

### Conflict of interest statement

The authors declare that the research was conducted in the absence of any commercial or financial relationships that could be construed as a potential conflict of interest.
